# Children’s psychological well-being and problem behavior during the COVID-19 pandemic: An online study during the lockdown period in Germany

**DOI:** 10.1371/journal.pone.0253473

**Published:** 2021-06-23

**Authors:** Natalie Christner, Samuel Essler, Astrid Hazzam, Markus Paulus

**Affiliations:** Department of Psychology, Ludwig-Maximilians-Universität München, Munich, Germany; Institute of Physiology and Basic Medicine, RUSSIAN FEDERATION

## Abstract

As COVID-19 dramatically changes human social life, restrictive lockdown periods to slow the spread of the virus have been suggested to particularly affect the psychological well-being of children and their families. To capture lockdown-related effects on a large scale, the present study used an online questionnaire completed by parents of 3-10-year-olds during the most restrictive lockdown period in Germany thus far (*N* = 2,672). Parents reported their stress level, their child’s well-being, and their child’s problem behaviors among others. Results showed that most parents and children experienced lockdown-related stress. Concerning children, not being able to meet with friends and family members outside the household emerged as the primary challenge. Older children (7–10 years) evidenced more emotional symptoms as well as less conduct problems and hyperactivity than younger children (3–6 years). Children’s own and their parents’ stress level, the degree to which children missed other children, and children’s age all showed to be negatively related to children’s general life satisfaction. Single parenthood and being an only child were associated with higher levels of child problems. Taken together, these findings shed light on the psychological well-being of children and their families during governmental lockdown measures, as well as on relations between children’s coping and demographic background. They have implications for possible avenues for interventions, inter alia by encouraging policies that facilitate the maintenance of social relationships and focus particularly on children from single parent families, on only children as well as on families in challenging housing situations.

## Introduction

The pandemic caused by the SARS-CoV-2 virus bears an enormous challenge for societies worldwide. In order to slow down the infection rate, many communities arranged lockdowns that involved extensive restrictions of public life. Events and gatherings were cancelled, shops and recreational facilities were closed, and employees had to work from home, if possible. In addition, educational institutions such as schools and universities were closed and began to move teaching online. Kindergartens and daycare centers only offered a reduced possibility of emergency child care for key workers (i.e., retail, health care system, banks, etc.). Later, emergency child care was expanded for single-parents. Besides these restrictions, governments recommend and enforce social distancing, that is, keeping physical distance from others, including friends and family members from different households. Hence, the COVID-19 pandemic massively curtails social interactions and public life.

Families are particularly affected by the contact restrictions and preventive regulations. First, parents’ working situation changed, potentially resulting in additional concerns about financial security. Some are required to work short time, some have to work from home, and some struggle with the maintenance of their own business. Others have to keep up their work, facing the threat of interacting with potentially infectious people every day. On top of that, parents’ responsibilities increased. Because most of the children had no access to kindergarten or school for weeks, they had to be taken care of the whole day, including teaching obligations. The accumulation of responsibilities thus likely constitutes a particularly stressful situation for parents and families [1]. The current study aimed to investigate the impact of the pandemic and associated restrictions on parents’ and children’s psychological well-being. In addition, we aimed to identify the major problems for children and factors that might attenuate the problematic consequences of the pandemic.

Following developmental theorizing, governmental restrictions should have a pronounced impact on young children. First, children are highly dependent on adults given their limited autonomy. That is, children rely on adults’ support in everyday tasks but also for emotion regulative processes. Given parents’ increased responsibilities, children might receive less or only inconsistent support. Second, young children might have problems to grasp the complexity of the situation and understand the massive changes in everyday life due to their limited cognitive abilities [2, 3], leading to perceived insecurity and lack of control. Third, due to their limited self-regulation and emotion regulation capacity [4, 5], children might be in special need for support in handling anxiety caused by the disruptive situation. The multitude of these conditions probably makes the situation particularly challenging for young children, which is hypothesized to lead to increased emotional and behavioral problems. First evidence during the COVID-19 pandemic supports this assumption [6–8].

A further line of reasoning leads to the assumption that health-promoting regulations caused by the COVID-19 pandemic had widespread effects on children’s life. Governmental measures restricted multiple systems in which–considering a bioecological framework [9]–children’s interactions are typically embedded, such as family, school, or daycare centers. As multiple systems are considered important to cope successfully with a challenging situation [10, 11], we have reason to assume that the pandemic-related regulations threaten children’s well-being. While children faced a challenging situation, which would require a successful resilience system, most resilience systems were temporarily disrupted. Children’s everyday life was concentrated on the family, likely leading to more pressure and stress within the family system.

First studies on the impact of the COVID-19 pandemic indeed evidence an increase of anxiety and depression symptoms among school-aged children [for review see 7]. In addition, research on previous pandemics revealed that disease-containment methods can have traumatizing effects. Following the Influenza-A-H1N1 pandemic, the proportion of children and parents meeting the clinical criterion for a posttraumatic stress disorder was higher in those who experienced isolation or quarantine compared to those who did not experience isolation or quarantine [12]. A review on the psychological consequences of quarantine in adults additionally highlights poorer mental health, increased fear, frustration, and a sense of isolation as consequences of quarantine [13]. The current study aimed to expand our understanding of pandemic-related stressors and protective factors for parents’ and children’s psychological well-being by identifying major problematic topics and examining relations with demographic background.

Given the intense pressure on the family system, caregiver stress might be passed on to children. From an attachment theoretical perspective, children seek proximity to caregivers when exposed to stressful situations [14, 15]. Given the stressful situation caused by the pandemic, parents experience more stress and, as a result, might provide less support to children than required. This leads to the special situation that children’s need for the presence and support of the primary attachment figure is heightened, while parents themselves face a stressful situation and hence struggle even more to meet the child’s needs adequately. These challenges for child emotion regulation might pave the way for developmental psychopathologies [e.g., 16, 17]. Thus, even though parents spent more time with children, they had possibly more problems in reacting to their children appropriately.

Although the lockdown regulations affected the family system immensely, the situation might have not only been perceived as stressful. Beyond the negative consequences, families might have perceived positive outcomes. Parents got to spend more time with their children, which might have led to valuable parent-child interactions. The interpretation of the lockdown situation in positive or negative terms might relate to the condition and demographic background of the family. It remains thus an open question to which degree families experienced positive and negative consequences of the lockdown.

The pandemic-related restrictions inhibit personal interaction with friends or other children (except for children living in the same household). This might be particularly challenging for children’s development, because peer relationships, particularly friendships, are important for several reasons. Friendships play an important role for children’s well-being, they provide reciprocal assistance and support children’s emerging emotion regulation abilities [18–21]. In addition, friendships offer children a context that is characterized by cooperation, prosocial behavior, and a strong affective tie [20]. For example, already preschoolers expect more sharing from friends than non-friends [22]. The lockdown-related isolation hinders direct interactions with friends. Particularly in younger children, friendship rests on joint activities such as playing together [23]. While older children might be able to maintain social exchange digitally, younger children are less capable in doing so. We therefore expect the contact restrictions to strongly affect children’s well-being.

Next to the social consequences of governmental restriction, the pandemic itself constitutes a psychological challenge for children. Fear of getting infected and concerns about the well-being of close others are potential causes of distress. The general ability to have empathic concern for others’ well-being emerges in the first years of life [24, 25]. In school years, children show anxiety about their own health [26, 27] and report fear of a novel disease, as evidenced during the Swine Flu pandemic [28]. These fears, particularly if not being adequately addressed by caregivers, could lead to maladaptive outcomes [17, 29]. It remains thus important to examine the degree to which children face a variety of concerns, in particular anxiety, in times of the pandemic.

Notably, the stressful situation might be detrimental for child well-being to such an extent that it relates to psychopathological outcomes. Developmental theories highlight the role of family or parental stress for the emergence of child problem behavior [30, 31]. A number of studies evidenced that parenting stress is associated with internalizing and externalizing problems in their children [32–34]. Because the situation caused by the pandemic is expected to be exceptionally straining for parents, changes in parenting behavior might in turn cause child problems [35]. In addition, anxiety of parents regarding the unpredictable situation might pave the way for internalizing disorders in children [36]. Examining children’s problems in times of the lockdown is thus of great importance to foresee potential psychopathological consequences of the governmental regulations for children.

While the considerations above suggest that the situation of the COVID-19 pandemic was particularly stressful for parents and children, it is possible that some families were less negatively affected than others, depending on the resources they had to cope with such a situation. For example, two-parent families might have been less affected than single-parent families. Single parents typically report more parental stress [37, 38], which–given reduced extra-familial resources during the lockdown–might have been even enhanced. The well-being of children with siblings might have been less affected than the well-being of only children because they had at least one other child to play with.

### Current study

The current study investigated the psychological impact of the contact restrictions and lockdown regulations during the COVID-19 pandemic on families and, in particular, young children aged 3–10. We focused on this age range because young children are very sensitive to the way of caregiving and highly dependent on their parents, given little autonomy and limited cognitive and emotional resources to handle the situation, as proposed above. These considerations give reason to assume that they might be particularly vulnerable to the changing pandemic-related conditions. In addition, the study aimed to identify the main topics that were relevant for children during the lockdown to identify possible avenues of intervention.

For that purpose, we assessed families’ situations during the most restrictive time of the COVID-19 pandemic (mid of April 2020) in an online study in a German sample. At the time of data acquisition, educational facilities and daycare centers were generally closed, only offering a minimal emergency child care for key workers. Meeting people from other households was prohibited and fined, and employees were mostly required to work from home, if possible. Temporary financial support was offered for parents, if they were not able to work because they had to take care of their child at home due to pandemic-related closing of institutions. Particularly single parents and families with low income were eligible for additional financial support. If required to work short-time, that is, being temporarily exempted from work and receiving a reduced payment because of the employer’s economic situation, parents received a larger continued payment than people without a child. The conglomeration of restrictions, which massively affected family life, called for an investigation of how children and parents handle this exceptional situation. In order to reach as many families as possible, we created an online questionnaire that included, inter alia, measures of parents’ and children’s stress level during the lockdown, changes in children’s quality of life, children’s problem behaviors, the extent to which children missed or engaged in social relationships, and pandemic-related challenges that were considered most problematic during the lockdown. Overall, we expected children’s quality of life to be negatively affected by the pandemic. Based on the theoretical considerations above, we predicted children’s change in well-being to be related to their own level of stress, to parental stress, and to decreased interaction with friends. In addition, we expected children to show problem behaviors, particularly if being an only child, living in a single parent family, or living in limiting housing amenities. We controlled for parental education when examining these relations. Yet, one needs to be aware that this does not fully control for socioeconomic status, making it possible that some of the associations might be related to families’ financial resources. In addition, we aimed to identify which topics were prevalent for children’s stress level, e.g., being concerned about an infection of themselves or close others, missing the interaction with other children, or being constantly surrounded by a parent, which might result in more disputes at home. The prevalence of such concerns might depend on the child’s age. With increasing age, friendships become more important, but children might also understand better why they cannot see their friends, that they are not alone in this, and that this situation will pass. With increasing age, children might also better understand the threat of a virus and therefore be more concerned about infections. We assessed the importance of a variety of concerns to shed light on their relative prevalence in children.

## Method

### Participants

A total of 2,672 participants made up the final sample. An additional 549 participants were excluded from the final sample as they did not fully complete the questionnaire (*n* = 474) or reported on children outside of the age range from 3 to 10 years (*n* = 75). As some participants entered data for more than one child (see procedure), we had parental reports of 3,389 children for parts of the analyses. Participant recruitment took place by postings on (social media) websites, by directly contacting families affiliated with the lab through Emails, and by words of mouth. Importantly, data collection took place from the end of April until the beginning of May 2020 when the lockdown restrictions were strictest in Germany to capture the situation of families and children during the most challenging time of the pandemic. The demographic characteristics of the sample are displayed in Table 1. Many participating families have a high socioeconomic status. Participants excluded due to incompleteness of responses did not differ from participants with complete responses with respect to their family status, single parenthood, and educational degree by more than 7 percentage points ((1) family status for complete vs. incomplete responders–married (80% vs. 76%), relationship & living together (13% vs. 18%), relationship but not living together (1% vs. 2%), divorced/separated, no relationship (4% vs. 3%), single (2% vs. 1%), widowed, no relationship (<1% vs. <1%); (2) single parenthood for complete vs. incomplete responders–no single parenthood (94% vs. 96%), single parenthood (6% vs. 4%); (3) Educational degree for complete vs. incomplete responders–university degree (50% vs. 46%), vocational training (23% vs. 30%), university of applied sciences degree (15% vs. 14%), professional academy (8% vs. 7%), master training (3% vs. 3%), no vocational degree (<1% vs. 1%)). In addition, participants could optionally enter their Email address for the purpose of taking part in a raffle of ten 50 € gift vouchers. The study was approved by the ethics committee of the Faculty of Psychology and Educational Sciences, LMU Munich, and constitutes the first report of an ongoing longitudinal project. Participants gave their online informed consent to taking part in the study.

**Table 1:** Demographic characteristics of the sample.

Demographic Variable	Subcategory	Percentage
Family Status	Married	80%
	Relationship & living together	13%
	Relationship but not living together	1%
	Divorced/separated, no relationship	4%
	Single	2%
	Widowed, no relationship	<1%
Single parenthood	No single parenthood	94%
	Single parenthood	6%
Educational degree	University degree	50%
	Vocational training	23%
	University of applied sciences degree	15%
	Professional academy	8%
	Master training	3%
	No vocational degree	<1%
Current job status	Home office	44%
	Job outside of the home	18%
	Parental leave	18%
	Reduced working hours	6%
	No job	4%
	Exempted	4%
	Other	6%
Additional childcare hours		
For Mothers	5–6 hours	38%
	7–8 hours	38%
	3–4 hours	11%
	9–10 hours	9%
	0–2 hours	4%
For Fathers	5–6 hours	27%
	7–8 hours	26%
	3–4 hours	23%
	9–10 hours	8%
	0–2 hours	16%
State of residence	Bavaria	70%
	North Rhine-Westphalia	7%
	Baden-Wurttemberg	6%
	Berlin	3%
	Other	<3%
Age child	3–6 years	67%
	7–10 years	33%
Educational institution child	Kindergarten	56%
	Elementary School	38%
	Pre-Kindergarten	5%
	None	1%

### Power analysis

We conducted a statistical power analysis in G*Power to calculate the required sample size. As there was no prior COVID-19-related research to rely on regarding the expected effect sizes and given the practical and theoretical importance, we aimed at detecting small to large effect sizes. Assuming alpha = .05 and power = .80 in a multiple regression analysis with 8 predictors, the projected total sample size was approximately *N* = 759. Therefore, our objective was a final sample of greater than *N* = 800.

### Materials

The online survey consisted of three parts: (1) demographics, (2) situation of the child during the COVID-19 pandemic and parental strategies, as well as (3) general measures of parental self-efficacy and parent-child relationship quality. The survey was completed by one parent. In the introduction of the survey, we asked participants that the parent who mainly cares for the child should complete the survey. In the context of the present study, we focused on a selection of measures and we will present these in the following.

#### Demographics

The demographic questions referred to information about the parent, the child, and the parental strain due to the COVID-19 pandemic. Concerning the parent, we assessed age and gender, family and partner status (married, in a relationship and living together, in a relationship and not living together, divorced or separated without partner, widowed, single), gender of partner (if applicable), number of children in the household, federal state of residence, housing situation (apartment or house, with or without balcony, no, small or large garden), highest educational degree (without, vocational degree, professional academy, master training, (applied) university degree) of self and partner (if applicable), current job status (no job, parental leave, home office, job outside of the home, reduced working hours, exempted, other) of self and partner (if applicable), percentage of childcare work relative to other caregivers (e.g., 80% of childcare work of study participant when the other caregivers account for 20% of childcare work). Concerning parental strain due to the COVID-19 pandemic, one question assessed how many more hours the parent cares for the child on a daily basis. In addition, three questions assessed whether the parent is strained to a greater degree due to the pandemic (e.g., “I am more stressed out in the current situation than normally”; Cronbach’s α = 0.91). A five-point Likert scale ranging from 1 (“do not agree at all”) to 5 (“totally agree”) was used to record parental responses. Concerning the child, demographic questions assessed age, gender, and educational institution (kindergarten, school).

#### Situation of the child during the COVID-19 pandemic

*Child’s strain*. One item assessed the degree to which children are stressed, irritated, or lonely with regard to the current situation on a 4-point scale (“To which degree is your child stressed, irritated, or lonely with regard to the current situation?”). The response scale ranged from 1 (“not at all stressed, irritated or lonely”) to 4 (“considerably stressed, irritated or lonely”).

*Changes in quality of life*. To assess how the shutdown during the pandemic affected the child, we adapted 12 items from the German translation of the 52-items KIDSCREEN Health-Related Quality of Life Questionnaire for Children and Adolescents [39]. We selected these specific items (see below) for three reasons. First, some scales were not applicable given social distancing during the lockdown (e.g., friends, school and learning, others). Second, some items of relevant scales were similar in wording and due to time constraints, we only included one item (e.g., “was in a good mood” but not “was happy”; “enjoyed life” but not “was satisfied with life”). Third, some scales were of more interest theoretically (e.g., feelings, general mood) and were thus included over others (e.g., physical activities and health). In the original version, the KIDSCREEN assesses children’s quality of life at a single time point. For the current purpose, we adapted the items in order to measure quality of life relatively to the time period before the lockdown and in order to measure positive and negative changes likewise (e.g., increase or decrease in quality of life compared to time period before the lockdown).

To answer the KIDSCREEN, parents indicated on a 7-point scale how much more or how much less their child had positive emotions, moods, time for him-/herself and with his/her parents during the weeks of the complete lockdown as compared to before the pandemic. The response scale ranged from 1 (“clearly less”) to 7 (“clearly more”) with the middle category 4 denoting “no difference”. The items were: (1) enjoyed life, (2) was in a good mood, (3) had fun (1–3 aggregated to scale “emotions”; Cronbach’s α = 0.89), (4) was sad, (5) felt so bad that s/he did not want to do anything, (6) was lonely (4–6 aggregated to scale “moods”; Cronbach’s α = 0.78), (7) was content (single item scale), (8) had time for her-/himself, (9) was able to do things s/he wanted to do in her/his free time (8–9 aggregated to scale “free time”; Cronbach’s α = 0.42), (10) felt that her/his parents had time for her/him, (11) felt fairly treated by her/his parents, and (12) has been able to talk to her/his parents when s/he wanted (10–12 aggregated to scale “family”; Cronbach’s α = 0.72). Due to its insufficient reliability, the scale “free time” was only used for descriptive purpose and excluded from the statistical analyses.

*Social relationships*. Five questions assessed how much the child missed his/her educational institution and friends, how often s/he asks about the reopening of kindergarten or school, whether s/he plays with others from different households (friends, children from the neighborhood, family members, child attends emergency group in kindergarten/school), and whether s/he initiates contact to her/his friends in any other way.

*Problem behaviors*. To get a more detailed insight into the child’s current behavior and well-being, we adapted three subscales (emotional symptoms, conduct problems, hyperactivity-inattention) of the Strengths and Difficulties Questionnaire [SDQ; 40]. Each subscale consists of 5 items and is answered on a 3-point scale (0 –“not true, 1 –“somewhat true”, 2 –“certainly true”). Other subscales were excluded as they largely refer to interactions with other children, which were almost non-existent due to the lockdown. Given the circumstances, the remaining items also had to be adapted and shortened (e.g., remove references to behavior at school or towards other children, which was not applicable during the lockdown). In order to keep the item structure similar, and in order to avoid ambiguous item formulations (e.g., “Often unhappy, depressed, or tearful”), we decided to adapt all items and to create short versions as follows: emotions problems (“Often complains of headaches”, “Has many worries”, “Often unhappy”, “Nervous or clingy”, “Has many fears”; Cronbach’s α = 0.78), conduct problems (“Often has temper tantrums”, “Generally obedient”, “Often fights”, “Often lies or cheats”, “Steals from home”; Cronbach’s α = 0.71), and hyperactivity (“Restless, overactive”, “Constantly fidgeting”, “Easily distracted”, “Reflects”, “Sees tasks through to the end”; Cronbach’s α = 0.65).

*Individual challenges*. Moreover, to assess the greatest challenges of the child during the lockdown, we provided 14 choices with multiple answers possible (e.g., “Child cannot meet its friends regularly”, “Child is bored”, “Conflicts about media usage”).

### Procedure

The online survey was hosted on Qualtrics and took participants approximately 15 minutes to complete. The instructions informed participants about the purpose of the study and participants agreed to data protection regulations.

Next, participants completed the three blocks of the survey in a fixed order. First, they completed questions on demographics and the strain experienced by parents during the lockdown. Second, they answered questions pertaining to the situation and well-being of the child and the parental strategies used. Third, they completed the parenting self-efficacy items and the parent-child relationship items. All questions were displayed as forced choice to minimize missing data. Throughout the survey, participants could navigate back and forth to change their answers if necessary.

In addition, participants had the opportunity to complete a shortened version of the survey for a second, third, fourth, and fifth child. This shortened version comprised four demographic questions (age, gender, educational institution, additional caregiving work because of the pandemic), the Kidscreen items and the problem behavior items as described above for the respective child (662 participants completed the survey for a second child, 54 participants completed the survey for a third child, and 1 participant completed the survey for a fourth child). At the very end, participants were thanked for their participation.

### Data coding

Numbers were assigned to the verbal markers of the scales as described above. We recoded all reverse items (3 items of the Kidscreen, 3 items on problem behaviors). Sum scores/mean scores were calculated for the respective scales and used for analyses. Data relevant for this study is openly available on OSF at https://osf.io/9bj23/.

### Data analysis

Sample sizes between analyses differ, because the questionnaire for a second, third, or fourth child of a family did not cover all variables. Therefore, some analyses focus only on one child per family (*N* = 2672) and some analyses focus on the total sample of children (possibly multiple children per family; *N* = 3389). In order to account for the large age range (3–10 years), we divided the sample for some analysis in children aged 3 to 6 years and children aged 7 to 10 years. This age split roughly represents preschool age and school age. In addition, we divided the sample for some analyses based on whether children come from a single parent family or no single parent family because single parents might be particularly burdened by contact restrictions. For these analyses, we excluded 30 children for whom it was unclear whether they come from single parent or not-single parent families (family status: “relationship but not living together”). Analyses were computed with R version 4.0.1.

## Results

### Stress of parents and children (*N* = 2672)

#### General stress level

**Fig 1:**
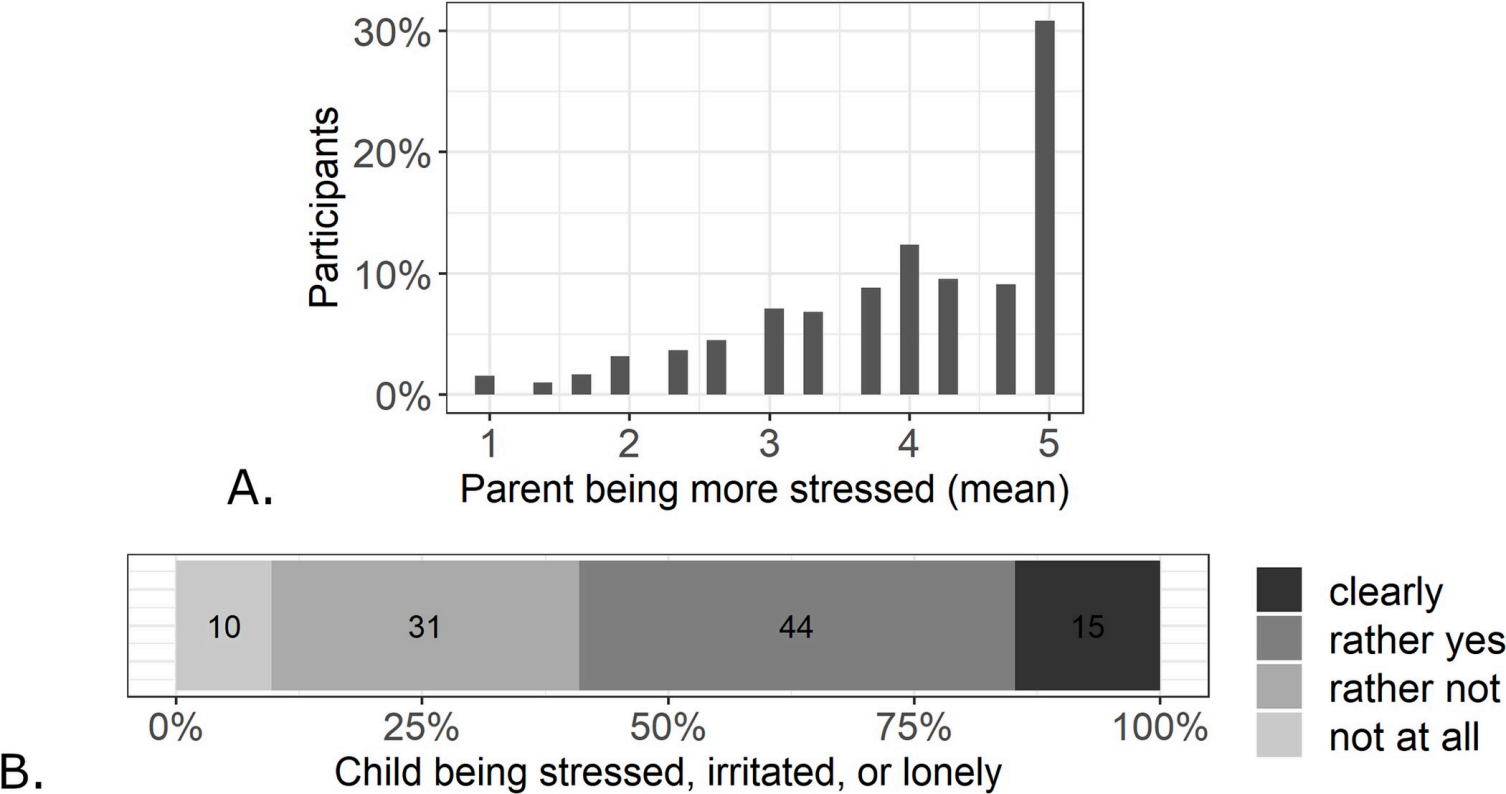
A: Percentage of parents’ mean ratings on items regarding current stress. B: Percentage of children who are reported to be stressed, irritated, or lonely with regard to the current situation.

Parents report to be more stressed than usual due to the current situation. 31% of the parents fully agreed with all three items that stated that the current situation is more challenging and stressful than usual. The frequency of parents’ mean rating across the three items about their stress level is displayed in Fig 1A.

The majority of children (>50%) were also reported to be rather or clearly stressed, irritated, or lonely with regard to the current situation (see Fig 1B). The mean level was identical for younger (*M* = 2.64, *SD* = 0.85) and older children (*M* = 2.64, *SD* = 0.85).

#### Reasons for stress in children

**Fig 2:**
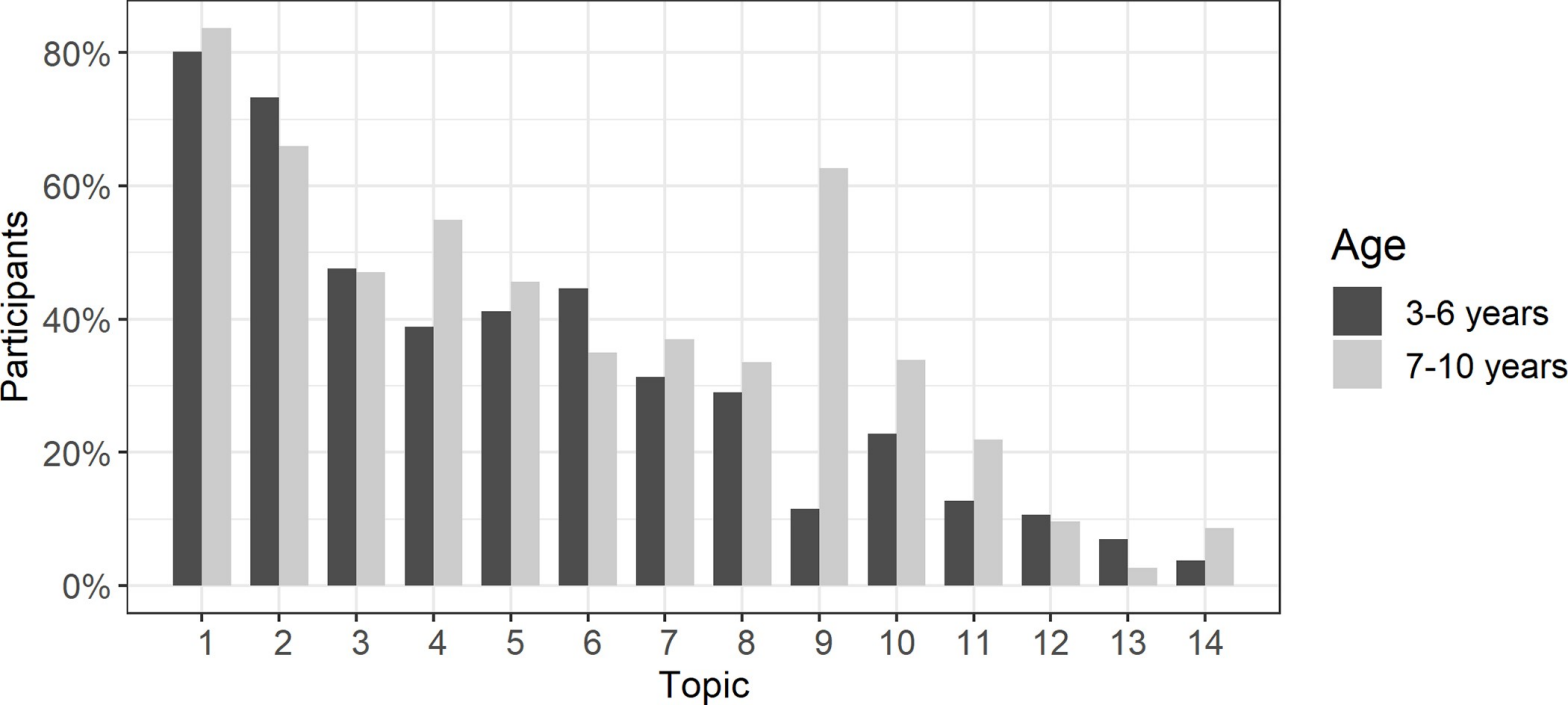
Reasons for the child being stressed, irritated or sad split by the child’s age. Topics: 1) Child cannot meet its friends regularly. 2) Child cannot meet other family members (e.g., grandparents). 3) Child is bored. 4) Child cannot engage in hobbies/sports. 5) Conflicts about media usage (e.g., mobile phone, computer, tablet). 6) Me or my partner are more irritable than usual and sometimes overreact. 7) More disputes with siblings. 8) Child cannot leave the apartment/house as he/she wants to. 9) Conflicts about doing schoolwork or other duties. 10) Conflicts about keeping a daily routine. 11) Concern that other people might get sick. 12) Child is constantly surrounded by one parent at home. 13) Others. 14) Concern about getting sick oneself.

The frequency of topics that are reported as being stressful for the child are displayed in Fig 2. For both preschool and school-aged children, the most prevalent topics are that they cannot meet their friends and other family members anymore. Particularly for school-aged children, disagreements about schoolwork or other duties and the lacking possibility of engaging in hobbies or sport are also reported frequently. Interestingly, what seems to be least problematic is children’s own fear of getting infected.

### Social relationships of children (*N* = 2672)

**Fig 3:**
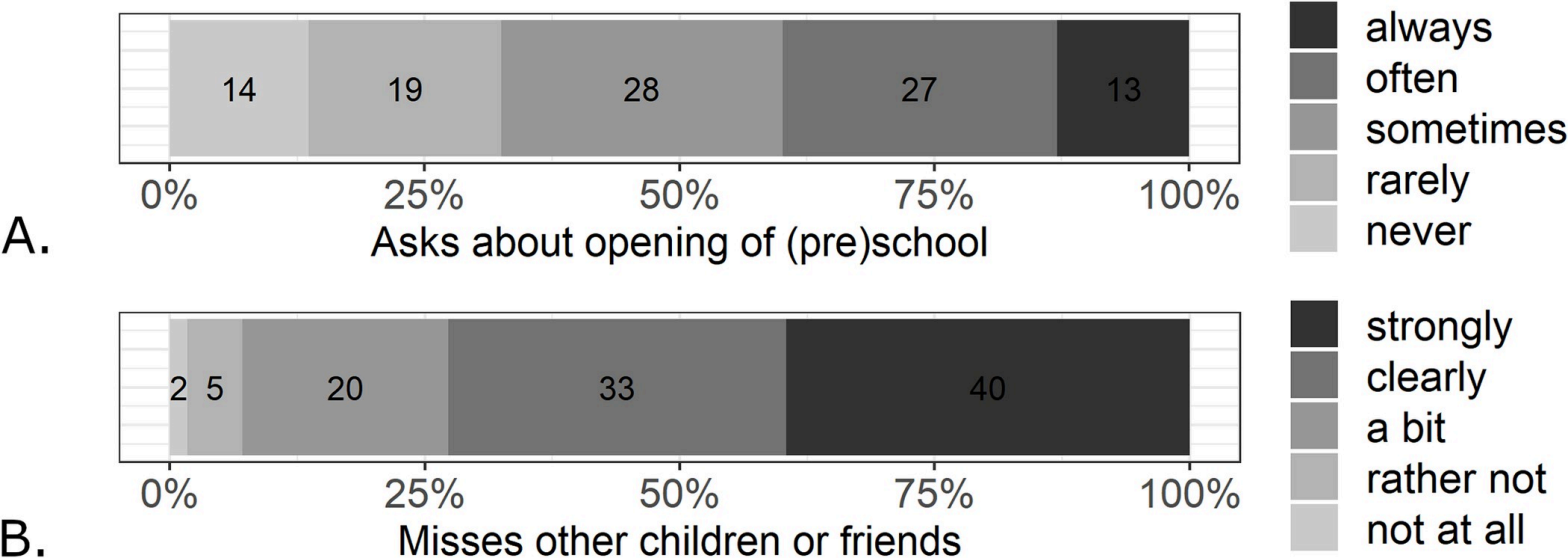
A: Frequency of children asking about the opening of their (pre)school. B: Intensity of children missing other children from their (pre)school or other friendships.

Due to the state-ordered restrictions, children were not able to visit (pre)school. Indicators of how much children miss their (pre)school and friends are displayed in Fig 3. The majority of preschool and school-aged children asked always, sometimes, or often when their preschool or school will open again (> 60%). The mean level was comparable in younger (*M* = 3.08, *SD* = 1.26) and older children (*M* = 3.05, *SD* = 1.17), *t*(2670) = 0.62, *p* = .533. In particular, more than 70% of parents indicate that their child misses other children or friendships clearly or strongly. This was stronger in older (*M* = 4.15, *SD* = 0.92) compared to younger children (*M* = 3.98, *SD* = 1.01), *t*(2670) = -4.07, *p* < .001.

### Contacts outside of the household (*N* = 2627)

**Fig 4:**
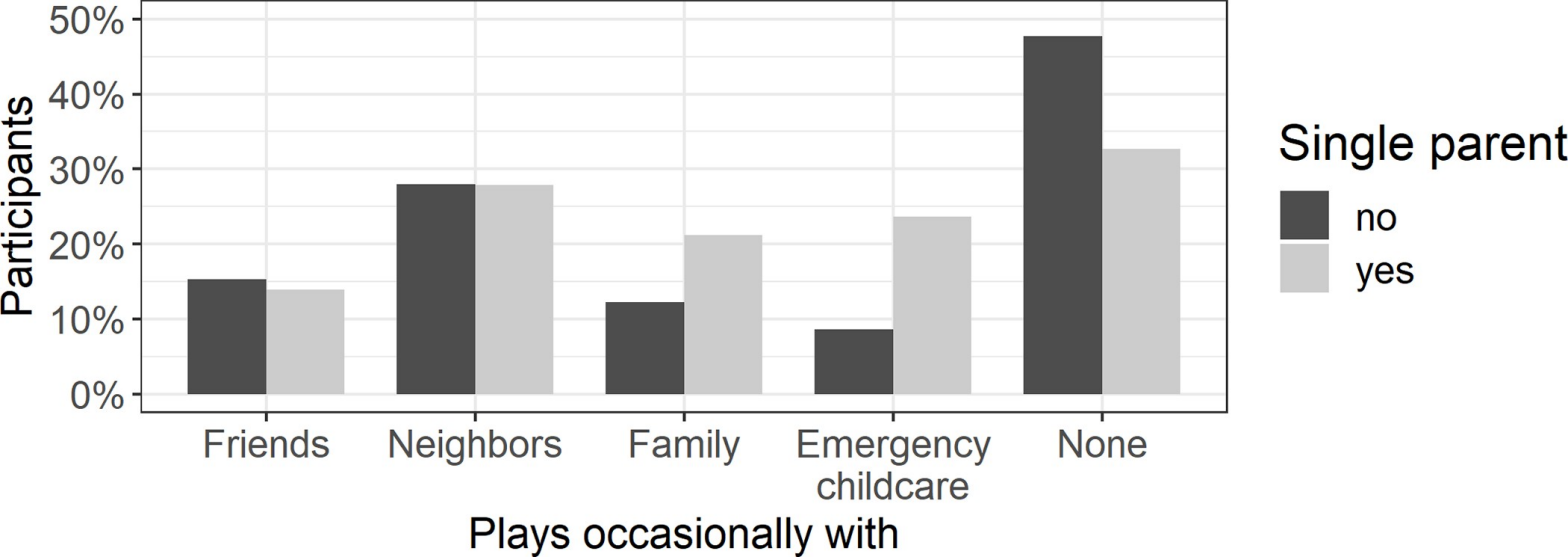
Percentage of parents reporting that their child plays occasionally with friends, neighbors, family members who lived in a different household, that their child occasionally visits the emergency childcare, or that nothing of the aforementioned applies. The percentages are split for single parents and non-single parents.

In order to examine children’s contacts outside of the household, we asked whether children played with others from different households (friends, neighbors, family members, attends emergency childcare groups) or whether nothing applied (multiple responses possible). For the following analysis, we excluded 15 children whose parent replied inconsistently to the question (selecting both “nothing applies” and one of the other response options). Overall, a number of families reported that their child played occasionally with friends (15%), neighbors (28%), or family members living in a different household (13%; see Fig 4). Independent of single parenthood, playing with neighbors was reported most often, if any answer was applicable. Particularly single parents reported that their child played with other family members.

### Children’s problem behaviors (*N* = 3352)

**Fig 5:**
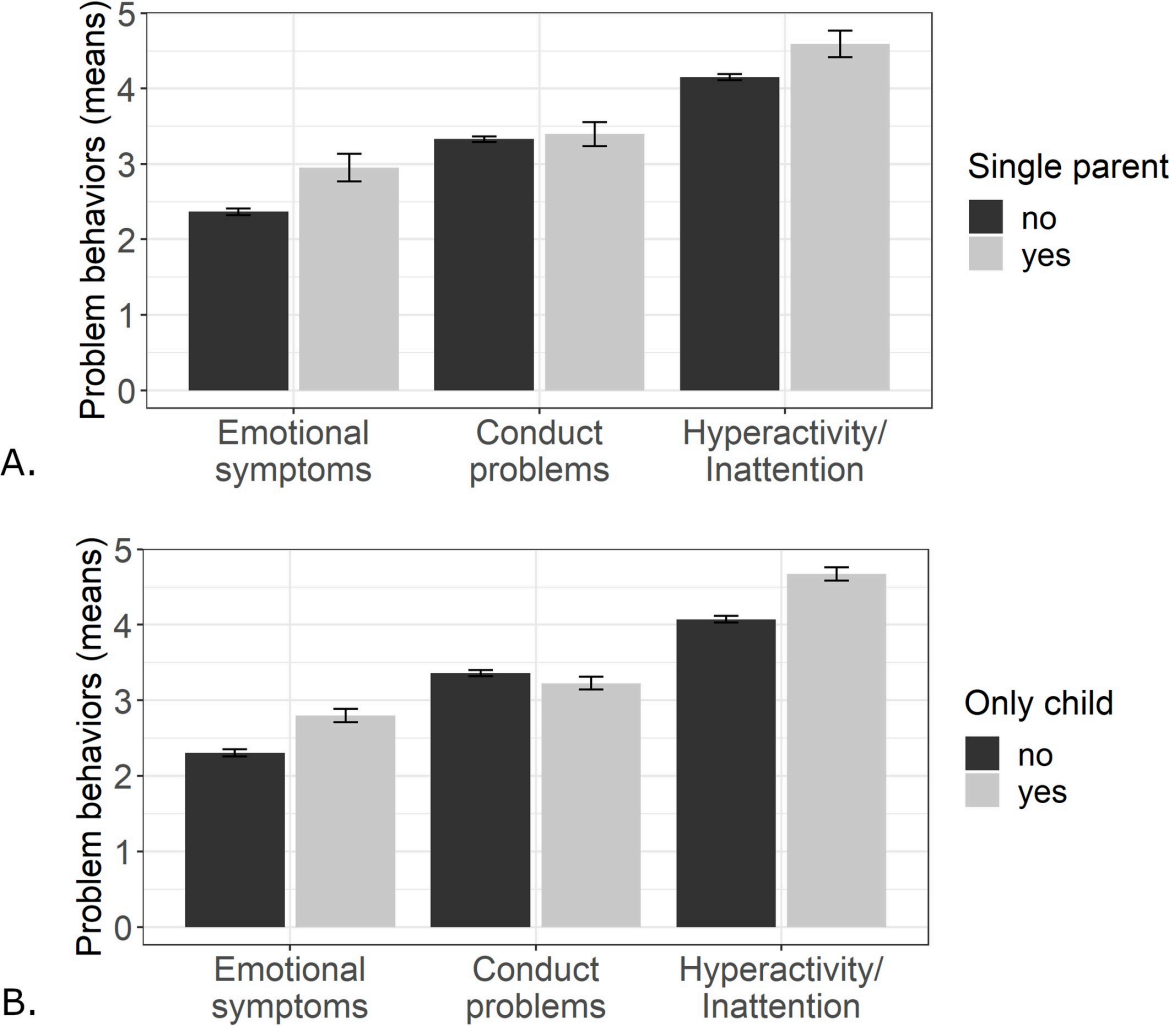
Means of problem behavior on each subscale across children. A: Children divided by their family background (single parent; not-single parent). B: Children divided by their sibling status (only child; not-only child).

We examined children’s problem behavior on three dimensions: emotional symptoms, conduct problems, and hyperactivity/inattention. In particular, we compared problem behavior between children coming from single parent (*n* = 197) and not-single parent families (*n* = 3159), and between only children (*n* = 651) and children with siblings (*n* = 2738). The mean sums across children on each subscale are displayed in Fig 5. We computed a multiple linear regressions for each scale, addressing the relation between coming from a single parent family or not and between being an only child or not and each problem behavior. We controlled for the age of the child, gender of the child, parental educational degree, and housing situation (apartment/house, balcony, small garden, large garden) by adding these variables as separate predictors. For these regressions, we excluded 10 children of diverse gender or for whom gender was not reported and 27 children whose caregiver replied ambiguously about their housing situation. The results of the regressions are presented in Table 2. Children from single parent families showed more emotional symptoms compared to children from not-single parent families. Likewise, only children showed more emotional symptoms and hyperactivity/inattention than children with siblings. Less hyperactivity/inattention was reported for children living in a house (*M* = 4.01, *SD* = 2.29) compared to children living in an apartment (*M* = 4.38, *SD* = 2.33). Children, who had a large garden at home, showed less hyperactivity/inattention (*M* = 3.93, *SD* = 2.27) and less conduct problems (*M* = 3.23, *SD* = 2.10) compared to children without a large garden (hyperactivity/inattention: *M* = 4.32, *SD* = 2.33; conduct problems: *M* = 3.38, *SD* = 2.15). Parental education related negatively to all aspects of children’s problem behavior.

**Table 2:** Linear regressions on children’s problem behavior.

	Emotional symptoms		Conduct problems		Hyperactivity/Inattention	
	*β*	*p*	*β*	*p*	*β*	*p*
Age	.05**	.004	-.11***	.000	-.08***	.000
Gender	.04*	.037	-.05**	.002	-.12***	.000
Parent education	-.07***	.000	-.09***	.000	-.13***	.000
House	-.01	.602	-.04^+^	.068	-.05*	.031
Balcony	-.03	.140	-.03^+^	.091	-.04^+^	.050
Small garden	.00	.854	-.03	.149	-.02	.293
Large garden	-.02	.413	-.05*	.046	-.07**	.001
Single parent	.04*	.034	.02	.394	.03	.111
Only child	.08***	.000	-.05**	.007	.07***	.000
*R*^*2*^, *p*	.02***	.000	.02***	.000	.05***	.000

*Note*. Gender: 0 = male, 1 = female. Parent education: 1 = none, 2 = vocational training, 3 = professional academy, 4 = master training, 5 = university of applied sciences degree, 6 = university degree. House: 0 = apartment, 1 = house. Balcony, small garden, large garden, single parent, only child: 0 = no, 1 = yes.

^+^ p < .10

* p < .05

** p < .01

*** p < .001.

### Changes in children’s quality of life (*N* = 3389)

**Fig 6:**
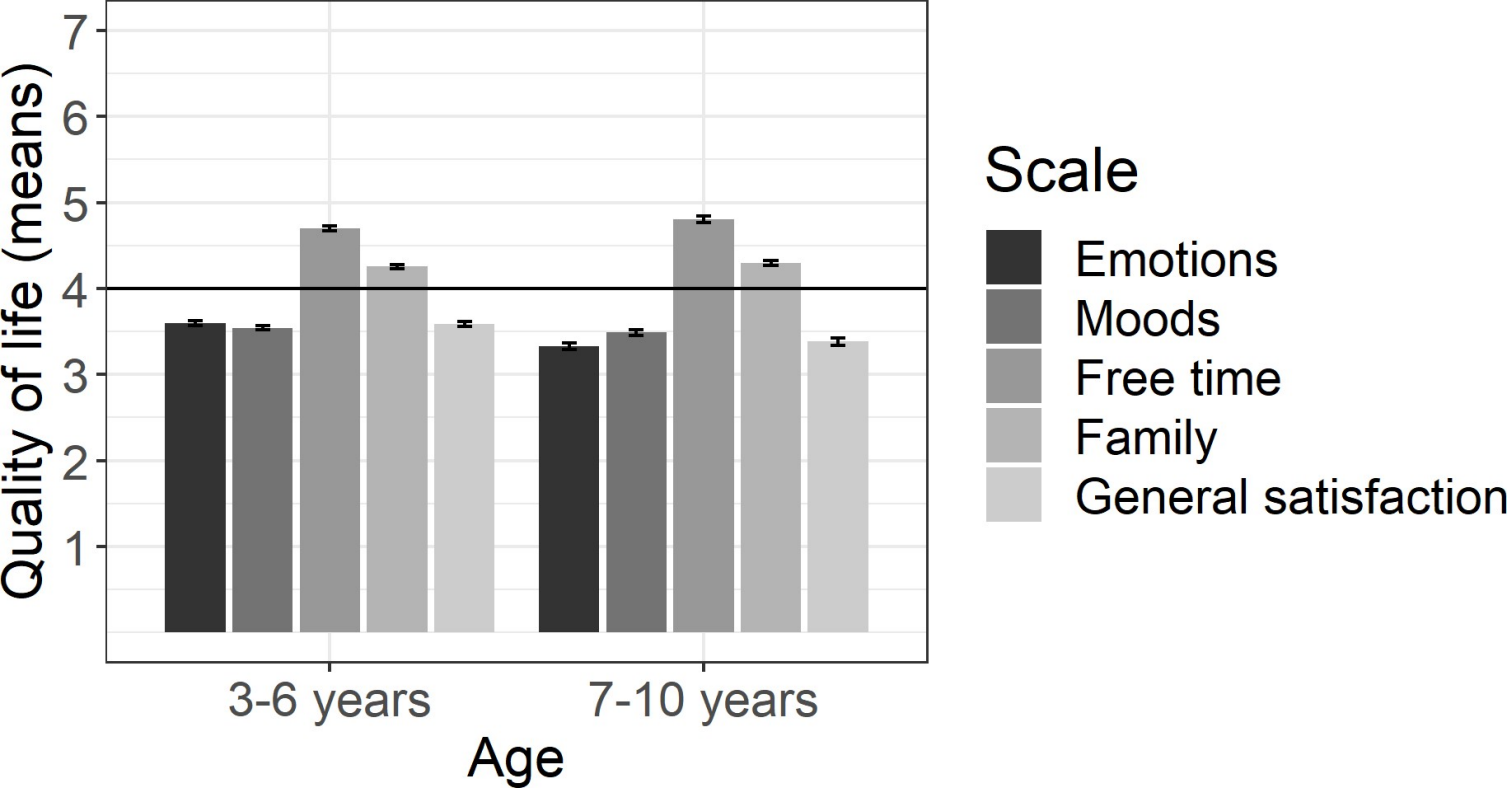
Changes in children’s situation in comparison to the situation before the pandemic and the associated restrictions split by children’s age. Error bars represent standard errors.

Fig 6 displays changes in children’s psychological well-being, moods and emotions, free time, family life, and children’s general satisfaction. A score of 4 indicates no change in comparison to the situation before the pandemic and the associated restrictions. We computed one-sample t-tests to compare means on each scale against 4. On the one hand, children’s emotions, moods, and their general satisfaction turned lower or more negative since the start of the pandemic and the associated restrictions, *p*s < .001, *d*s range from 0.35–0.41. On the other hand, children’s free time and family life turned more positive, *p*s < .001, *d*s range from 0.24–0.54.

We computed a multiple linear regression on children’s general satisfaction in order to investigate which factors mainly contribute to children’s well-being (*N* = 2672). For that purpose, we considered children’s age, children’s stress level, parental increase in stress level, children’s missing of other children or friends, and whether children had contact to people outside the household as predictors. Table 3 presents the regression results. Children’s general satisfaction related negatively to children’s stress level, parental stress level, and the level children missed other children or friends. In addition, the results show a small relation between children’s age and general satisfaction. With increasing age, children were less satisfied.

**Table 3:** Linear regressions on children’s general satisfaction.

	General satisfaction	
	*β*	*p*
Age	-.04	.004
Stress level	-.39	.000
Parental increase in stress	-.19	.000
Missing other children	-.19	.000
Contact to others	-.01	.514
R^2^, p	.42	.000

*Note*. Contact to others: 0 = no, 1 = yes (any of playing with friends, neighbors, family members outside household, emergency childcare).

## Discussion

The present study aimed to uncover the situation of children and their families during the most restrictive lockdown period of COVID-19 thus far. By means of an online questionnaire, parents reported on their own and their children’s well-being, stress experiences, and demographic characteristics. Results showed that the majority of both, parents and children, experienced lockdown-related stress. For children, not being able to meet with friends and family members outside the household presented the most challenging aspect of the lockdown. Older children evidenced more emotional symptoms as well as less conduct problems and hyperactivity than younger children. Single parenthood and being an only child were associated with more child problems. These findings highlight the effects of lockdown measures on families during COVID-19 and add to the growing body of studies showing similar findings in different countries [41–43].

Our findings highlight family challenges during the lockdown. In detail, parents reported more hyperactivity and conduct problems for younger as compared to older children. One should be aware that our results could mirror normative age-related changes. Previous research has shown normative age-related decreases in hyperactivity and conduct problems for girls and increases in emotional symptoms for boys and girls [44]. Given that we lack a direct comparison with the situation before the lockdown, we cannot quantify to which extent the difficulties reflect normative age-related changes and to which extent they are related to the pandemic restrictions. Considering a relevance of the restrictions, age differences might be related to the yet limited self-regulative capacities of younger children [5]. Younger children might have been especially challenged by adapting to a less structured life at home, as preschools could rely less on online teaching than schools and many parents were highly involved in restructuring their professional duties. Notably, older children showed more emotional problems than younger children. It is possible that older children might have been more emotionally challenged by the absence of their friends, who play an important role in the emotional well-being and regulation in middle childhood [20, 21]. Although we need to be cautious in our interpretation of this set of variables, they offer an overview on the challenges with which families had to deal and they provide the background for the interpretation of children’s and parents’ stress during the lockdown period.

Importantly, limited or non-existent contact with other children, friends, and the extended family seems to be one of the most critical factors for children’s stress during the lockdown. This becomes evident on multiple layers. Parents reported missing other children and family members as the main reason for children’s stress and indicated that the majority of children frequently asked about reopening of (pre)school. The seriousness is further underscored by one third of parents reporting that their child occasionally played with children from other households, thereby violating governmental policies. These findings speak to theories highlighting the importance of the mesosystem (extended family, peers, neighbors etc.) for child well-being [9]. They also underline theoretical considerations about contacts outside the core family as a resilience factor [45] and point to peer relationships as a crucial factor in children’s abilities to cope with social upheaval induced by COVID-19.

With regard to sociodemographic factors, the current findings reveal a negative relation between parental education level and all aspects of child problem behaviors. Previous research on this relation provides mixed evidence, with some studies showing negative relations between maternal or paternal education and child problem behaviors [46–48], and others suggesting only a relation with hyperactivity/inattention [49] or no relation with changes in externalizing or internalizing behaviors across middle childhood [50]. While the current study suggests that particularly children of parents with relatively lower education expressed problems, these findings might partly reflect a general pattern, irrespective of the pandemic.

Another finding suggests that single parenthood is associated with children’s well-being during the lockdown, especially concerning children’s emotional state. However, as previous research revealed that children from divorced families generally show more internalizing and externalizing behavior problems [51, 52], we have to be cautious in attributing these differences particularly to the lockdown situation. Nevertheless, these findings highlight the special challenge for single-parent families. This becomes also evident in the finding that particularly children from single parents met family members from different households. Due to the very limited access to extra-familiar resources, such as caretaking arrangements and interactions with friends or neighbors during the lockdown, single parents might have been faced with greater challenges concerning childcare, financial insecurity, or workplace reorganization than not-single parents. In addition, as findings indicate that single parents in general experience more stress than two-parent families [37, 38], this might have made them more vulnerable during the lockdown in the first place. Thus, our study suggests that targeting single parents during COVID-19 related measures could be a promising avenue for interventions to ensure child and family well-being. For example, one could argue that children of single parents should be prioritized in the allocation of emergency child care slots.

Beyond single parenthood, sibling status was related to child problems during the lockdown. Compared with children having siblings, only children have been reported to show more emotional and hyperactivity problems but less conduct problems. Previous research suggests that only children and children with siblings do not differ in means of adjustment or mental health [53, 54]. We might thus conclude that only children show increased emotional and hyperactivity problems compared to children with siblings particularly in the lockdown situation. If one considers this finding along with the finding that the most stressful aspect of the lockdown was for children to not meet friends and extended family, only children could have experienced a culmination of social isolation. That is, while children with siblings might have been able to compensate for their absent friends by engaging more intensively with their siblings, only children were devoid of any peer contact. Thus, sibling status seems to be associated with child well-being as far as social distancing measures are concerned.

A further factor that relates to children’s problem behaviors is their housing situation. Particularly the availability of a balcony or a large garden seems to be negatively linked with hyperactivity and conduct problems. Living in a house compared to an apartment seems to be linked with less hyperactivity. Research on housing characteristics and well-being suggests that children’s problem behaviors generally relate to housing quality [55] and housing type (high-rise-dwelling vs. low-rise- or house-dwelling, [56]). Other studies on housing situations report neighborhood effects with increased externalizing problems in children living with low-SES neighbors [57]. Since we lack a systematic comparison with the situation before the lockdown, we have to be cautious in concluding that relations between housing characteristics and problem behaviors are specific to the pandemic. Moreover, it is possible that these relations are attributable to families’ financial situation. We did not control for parents’ socioeconomic status, only for parental education, which can serve as one indicator thereof [e.g., 48, 58]. Therefore, associations with household amenities may pick up relations between family financial resources and child problems.

Apart from identifying factors that relate to children’s well-being, our results suggest that the lockdown has not only negative consequences for children and their families. Specifically, children were reported to have more family satisfaction than before the pandemic. During the lockdown, children may have valued the additional time spent with their parents, which possibly led to more family satisfaction. That is, if the family system had enough resources, children and parents could meaningfully engage with one another apart from external social duties. This positive consequence might have been especially pronounced if parent-child relationship quality was high at the beginning of the pandemic. Thus, our findings suggest that there may be positive side-effects of the lockdown. We have to leave it to future research to study these effects in more detail.

The current findings allow for some policy implications. As outlined above, the reduced possibility to meet friends and family members emerged as a dominant factor for children’s well-being. Although social distancing measures are required for slowing the spread of the virus [59], these findings suggest that policy measures should try to facilitate social relationships nevertheless. Allowing to meet other children and family members in compliance with hygiene regulations might be a suitable strategy to balance children’s need for social contact, parents’ need for external support, and pandemic-related preventive measures. Beyond that, the findings encourage policy measures that focus particularly on children from single parent families, only children, and children from constrained household amenities. Because higher levels of problem behaviors are reported, offering the limited number of emergency child care slots for these children might be a beneficial strategy.

### Limitations and conclusion

Although our study contributes to our understanding of the psychological context as well as the consequences of the pandemic for young children, it also comes with a number of limitations. First, we relied on parental report measures to get insights into the family dynamics during the COVID-19 pandemic. These might be particularly limited to assess children’s emotional experiences. While there were few other options for large-scale data collection during the lockdown, as personal contact was prohibited, future research should employ more direct methods to assess children’s behavior and experience during these challenging times. Second, our sample constitutes a convenience sample in which parents of high socioeconomic status are overrepresented, which decreases the external validity of the present study. This sample bias might result from the online questionnaire format, which requires an environment with technical devices and good internet access. Although there are also a number of families from low socioeconomic background, more research is needed to more accurately determine pandemic related effects across the socioeconomic scale. Moreover, as we did not control for families’ socioeconomic status, relations between child outcomes and household amenities may pick up relations with families’ financial resources. Third, our study is limited to the age range from young to middle childhood. It would be very insightful to see how older children and adolescents were able to cope with the pandemic and which different difficulties emerge across the span of childhood and adolescents. Fourth, the current study examined one time point during the pandemic. As a direct comparison with the situation prior to the pandemic is lacking, we cannot determine to which degree relations of demographic variables with problem behaviors and well-being are specific to the pandemic. In addition, longitudinal studies that examine parents’ and children’s well-being on several time points are needed to understand long-lasting effects of the lockdown measures and longitudinal relations between parent and child well-being.

Taken together, our study documents the psychological well-being and problems of children and families during the strictest COVID-19 related lockdown so far. In particular, both parents and children experience high levels of stress, with parental stress constituting one avenue to the reported internalizing and externalizing problems in children. While several demographic variables seem to relate to how families and children cope with the pandemic, the most important ones seem to be parent status (single, not single parent) and sibling status (only child, not only child). With social isolation as the major factor in children’s pandemic-related stress, there also seem to be singular positive effects regarding family life. Thus, our study can speak to public policy measures and interventions targeting family well-being during the unfolding COVID-19 pandemic.
